# RAA-CRISPR/Cas12a-Mediated Rapid, Sensitive, and Onsite Detection of Newcastle Disease in Pigeons

**DOI:** 10.3390/vetsci11100473

**Published:** 2024-10-04

**Authors:** Libin Liang, Dou Wang, Zhen Gao, Jiao Tang, Xing Li, Pengfei Ren, Ying Wang, Shimin Gao, Xingchen Wu, Yanna Guo, Bo Yang, Junping Li

**Affiliations:** College of Veterinary Medicine, Shanxi Agricultural University, Jinzhong 030801, China; lianglibin@sxau.edu.cn (L.L.); z20213673@stu.sxau.edu.cn (D.W.); z20223796@stu.sxau.edu.cn (Z.G.); z20223813@stu.sxau.edu.cn (J.T.); s20222402@stu.sxau.edu.cn (X.L.); t20225025@stu.sxau.edu.cn (P.R.); wangying@sxau.edu.cn (Y.W.); smgao@sxau.edu.cn (S.G.); wuxingchen@sxau.edu.cn (X.W.); gyn13655183568@163.com (Y.G.); bo_yang@sxau.edu.cn (B.Y.)

**Keywords:** pigeon Newcastle disease, RAA, CRISPR/Cas12a, detection

## Abstract

**Simple Summary:**

Pigeon Newcastle disease is a severe infectious disease in pigeons, characterized by a high infection rate and mortality rate. Quick and accurate detection of this disease is crucial for preventing outbreaks. In our research, we developed a quick and accurate method to diagnose this disease using a combination of two advanced techniques: recombinase-aided amplification (RAA) and CRISPR/Cas12a. This method first amplifies parts of the virus’s genetic material and then detects it, allowing for simple visualization using a lateral flow dipstick (LFD). Our tests showed that this method is very specific, meaning it does not react with other viruses, and it can detect very low amounts of the virus. When tested on real clinical samples, our new approach was performed, as well as the current gold standard test, quantitative real-time PCR. This new approach provides an important technical means for the rapid diagnosis of pigeon Newcastle disease onsite, enabling the timely prevention and control of Newcastle disease outbreaks.

**Abstract:**

Pigeon Newcastle disease, caused by pigeon paramyxovirus type 1 (PPMV-1), is a significant infectious disease in pigeons that can result in substantial mortality and poses a severe threat to the pigeon industry. The rapid and accurate onsite diagnosis of pigeon disease is crucial for timely diagnosis and the implementation of effective prevention and control measures. In this study, we established a rapid detection method for PPMV-1 based on recombinase-aided amplification (RAA) and CRISPR/Cas12a. The RAA primers target the conserved regions of the L gene for preamplification in clinical nucleic acid samples, followed by CRISPR/Cas12a detection of the target gene. Visualization could be achieved by combination with a lateral flow dipstick (LFD). This method demonstrated high specificity, showing no cross-reactivity with non-PPMV-1 samples. The sensitivity of the method assessed by fluorescence analysis reached 10^0^ copies/µL, and when it was combined with an LFD, the sensitivity was 10^3^ copies/µL. The constructed RAA-CRISPR/Cas12a-LFD visual detection method was applied to clinical sample testing and was found to enable the rapid and accurate detection of swab samples and tissue specimens. Its sensitivity was consistent with the current gold standard, quantitative real-time PCR results. The RAA-CRISPR/Cas12a-LFD detection method we developed provides a novel approach for the rapid, simple, precise, and specific onsite diagnosis of pigeon Newcastle disease.

## 1. Introduction

Newcastle disease (ND) is an important contagious disease caused by the Newcastle disease virus (NDV), which affects the majority of avian species and causes significant economic losses to the poultry industry worldwide [1]. Pigeon paramyxovirus type 1 (PPMV-1) is an antigenic variant strain of NDV that primarily infects pigeons, leading to Newcastle disease in pigeons and posing a significant threat to the pigeon breeding industry [2,3,4]. Currently, PPMV-1 is prevalent in many countries and regions worldwide. In China, several provinces have reported the presence of PPMV-1 infections in pigeon flocks, causing severe harm to both meat pigeons and racing pigeons [5,6].

Diagnostic methods for Newcastle disease in pigeons, specifically PPMV-1 infection, include virus isolation, hemagglutination (HA) and hemagglutination inhibition (HI), and reverse transcription–polymerase chain reaction (RT–PCR) [5,7,8]. However, these detection methods have limitations, such as long processing times, the requirement for expensive equipment, and inconvenience for onsite testing. Therefore, a rapid, precise, and specific PPMV-1 detection method that is convenient for onsite application is highly important for the prevention and control of pigeon Newcastle disease.

The clustered, regularly interspaced short palindromic repeats (CRISPRs) system was first discovered in the 1980s and has emerged as a preferred tool for genome editing [9,10,11]. In recent years, the CRISPR/Cas system has been widely developed for onsite rapid pathogen detection due to its exceptional specificity and sensitivity. This method relies on the nonspecific nuclease activity elicited by the Cas12a or Cas13a protein upon binding to the target gene sequence guided by gRNA [12,13,14]. To date, the combination of isothermal amplification technology (IAT) and CRISPR/Cas12a has been applied to detect pathogens such as SARS-CoV-2, the Monkey B virus, human norovirus, and ASFV [15,16,17,18]. In the field of avian disease research, a recent study demonstrated that the combination of Cas13a and lateral flow dipstick (LFD) can rapidly detect H5 avian influenza virus (AIV), offering a more convenient and precise method for detecting highly pathogenic avian influenza [19]. In this study, we developed a novel detection method that combines CRISPR/Cas12a with recombinase-aided amplification (RAA) for the identification of PPMV-1. This assay involves three steps: (1) an RNA extraction and RT-RAA reaction at 42 °C for 30 min; (2) CRISPR/Cas12a reaction at 37 °C for 10 min; and (3) rapid detection within 2 min via a fluorescence analyzer or LFD. Our novel molecular detection approach (RAA-CRISPR/Cas12a-LFD) provides a rapid, precise, and specific way to detect PPMV-1 without the need for sophisticated equipment. It is well suited for the swift screening and diagnosis of clinical samples, offering vital technical assistance for prompt confirmation and the efficient prevention and control of pigeon Newcastle disease.

## 2. Materials and Methods

### 2.1. Virus and Clinical Samples

Avian influenza virus (AIV), pigeon circovirus (PiCV), and avian infectious bronchitis virus (IBV) strains were preserved by the Laboratory for Prevention and Control of Major Poultry Diseases (Shanxi Agricultural University, Jinzhong, China). In this study, swab samples were collected from the throats and cloacas of meat pigeons at live bird markets and pigeon farms in Jinzhong City, Shanxi Province, China. A total of 126 swab samples were collected from live bird markets, while 60 swab samples and 15 tissue samples were gathered from pigeon farms. Viral nucleic acids were extracted using the 8-Minute Superfast Virus DNA/RNA Extraction Kit (Zhongshi Gene Technology Co., Ltd., Tianjin, China), with the operation process conducted with strict adherence to the manufacturer’s instructions. The viral cDNA used for qPCR and PCR was generated via the reverse transcription of the viral genomic RNA using the ReverTra Ace^TM^ qPCR RT Kit (TOYOBO Co., Ltd., Shanghai, China). The viral genomic RNA/cDNA was stored at −80 °C until use.

### 2.2. Reagents and Instruments

The HiScribe™ T7 Quick High Yield RNA Synthesis Kit, En Gen^®^ Lba Cas12a, and Buffer 2.1 were purchased from New England Biolabs (Ipswich, MA, USA). The Spin Column RNA Cleanup & Concentration Kit was acquired from Sangon Biotech (Shanghai Co., Ltd., Shanghai, China). PrimeSTAR Max Premix (2X) and the reporter probe were obtained from Takara Bio (Dalian, China). The RT-RAA Basic Nucleic Acid Amplification Kit was obtained from Hangzhou ZC Bio-Sci & Tech Co., Ltd. (Hangzhou, China). CRISPR Cas12/13 HybriDetect strips were purchased from Warbio Biotech (Nanjing, China). The handheld electric tissue grinder (TGrinder) and Universal DNA Purification Kit were purchased from Tiangen Biotech Co., Ltd. (Beijing, China). RT–qPCR was conducted in QuantStudio 5 (Applied Biosystems, Foster City, CA, USA).

### 2.3. Design and Synthesis of CRISPR RNA (crRNA) and Target DNA Primers

The full-genome sequences of 28 PPMV-1 strains were obtained from the NCBI database and aligned using MegAlign software (version v 7.1.0). On the basis of the nucleotide recognition properties of CRISPR/Cas12a (5′-TTTN-3′) and the results of genome sequence alignment, a PAM sequence was chosen at the relatively conserved L gene of PPMV-1. A pair of crRNA transcription template strands was designed, incorporating the T7 promoter, the scaffold sequence of LbCas12a, and the 20 bp sequence adjacent to the PAM sequence as essential reference elements. A portion of the gene sequence that contained the conserved region of the L gene was selected as the target gene sequence. The crRNA transcription template strands and target DNA primers are presented in Table 1. The two synthetic crRNA transcription template strands were gradually annealed to form a double strand following denaturation. In vitro transcription and purification were subsequently performed using the HiScribe™ T7 Quick High Yield RNA Synthesis Kit and a column-based RNA rapid concentration and purification kit, respectively.

### 2.4. ssDNA Reporter Probe Design and Validation

The reporter probe, consisting of a labeled fluorescent group (FAM), a quenching group (BHQ1/Biotin), and the sequence TTATTATT, was synthesized by TaKaRa Bio (Dalian, China). The synthesized reporter probe was diluted with enzyme-free water to a concentration of 200 μM to prepare a stock solution. The stock solution was further diluted to a final concentration of 1 μM in a light-protected tube to form the working solution, with the remainder stored at −20 °C until use.

To evaluate the trans-cleavage activity of CRISPR/Cas12a, a PCR tube was prepared with a total volume of 20 μL, containing 2 μL of Cas12a (1 μM), 1 μL of crRNA (100 ng/μL), 2 μL of buffer, 1 μL of RNasin (50 U), 10 μL of RNase-free ddH_2_O, 3 μL of target DNA, and 1 μL of Reporter I (1 μM) (5′ FAM-TTATTATT-BHQ1 3′). The mixture was subsequently analyzed via an QuantStudio 5 instrument to detect the trans-cleavage activity of CRISPR/Cas12a.

### 2.5. Primer Design and Recombinase-Aided Amplification (RAA)

A set of primers was designed on the basis of the detection site of PPMV-1 and the principles of RAA primer design. After a series of RAA primers were tested, the one with the best amplification effect was chosen for subsequent experiments. The sequences of the primers used were as follows: PPMV-1-RAA-F: AACCTCAACTAACCGCCTCTTGATAGAGTTT and PPMV-1-RAA-R: CTGCCATTACCTGGCAGTTTCTTAATCT. The lyophilized primers were diluted to 10 μM with enzyme-free water to prepare the working solution, and RAA was carried out according to the instructions of the RT-RAA Basic Nucleic Acid Amplification Kit.

### 2.6. RAA-CRISPR/Cas12a Sensitivity Analysis

The amplified and recovered product of the target DNA was diluted to concentrations ranging from 10^0^ to 10^10^ copies/μL, and these dilutions were then used as templates for subsequent reactions after amplification with the RT-RAA Basic Nucleic Acid Amplification Kit. Sensitivity analysis was conducted using the QuantStudio5 FAM channel to collect fluorescence signals every 2 min, with three replicates performed for each reaction. The reaction protocol consisted of a pre-reaction step at 37 °C for 1 min, followed by fluorescence signal acquisition at 37 °C for 2 min (30 cycles).

### 2.7. RAA-CRISPR/Cas12a Specificity Analysis

To evaluate the specificity of the established RAA-CRISPR/Cas12a method for targeting PPMV-1, we selected AIV, IBV, and PiCV for testing. Following viral RNA amplification via RT-RAA, the reaction mixture was prepared with 2 μL of Cas12a (1 μM), 1 μL of crRNA (100 ng/μL), 2 μL of buffer, 1 μL of RNasin (50 U), 10 μL of RNase-free ddH_2_O, 1 μL of Reporter I (1 μM), and 3 μL of the RAA-amplified reaction mixture containing viral RNAs or ddH_2_O as a negative control. The fluorescence signals were detected at 2 min intervals via the QuantStudio 5 FAM channel, with each reaction performed in triplicate.

### 2.8. RAA-CRISPR/Cas12a-LFD

To visualize the analysis results, we established an LFD-based RAA-CRISPR/Cas12a assay. The reaction mixture was assembled with 2 μL of Cas12a (1 μM), 1 μL of crRNA (100 ng/μL), 2 μL of buffer, 1 μL of RNasin (50 U), 10 μL of RNase-free ddH_2_O, 1 μL of Reporter II (5′ FAM-TTATTATT-biotin 3′) (1 μM), and 3 μL of the recovered target DNA product after RAA. The reaction mixture was incubated at 37 °C for 10 min, and then the volume was adjusted to 60 μL with RNase-free water, followed by thorough mixing. The dedicated Cas12/13 nucleic acid test strips were subsequently inserted into the reaction tube. The strips were allowed to saturate the entire reading area, which typically took 1–2 min. The results were directly read on the basis of the coloration of the test strips. The presence of red bands at both the control line (C line) and the test line (T line), or the absence of coloration at the C line while the T line showed a red band, indicated that the reporting group was activated, and both scenarios are considered positive results. Conversely, if only the C line displays a red band while the T line does not, the result is interpreted as negative.

### 2.9. Clinical Sample Testing

Sixty pigeon throat and cloacal swab samples were collected from live bird markets and pigeon farms and preserved in PBS. Three positive tissue samples originating from a pigeon farm were pulverized using liquid nitrogen and subsequently suspended in PBS. Both the 60 pigeon swab samples and the 3 positive tissue samples were homogenized via a vortex mixer and then centrifuged at 10,000 r/min for 10 min at 4 °C. Nucleic acids were extracted from the samples via an 8 min ultrafast virus DNA/RNA dual extraction kit. The detection of PPMV-1 was performed via the RAA-CRISPR/Cas12a-LFD assay, with real-time quantitative PCR (qPCR), and a conventional PCR was also employed for comparative analysis. The primer sequences for qPCR were as follows: PPMV1-F: 5′-GCAGGATACAAGGATCTG-3′; PPMV1-R: 5′-GCTACACTGCCTATAATG-3′; and the PPMV1-Probe: 5′-FAM-TTCTGCCTCCTTCCTCCTGATG-BHQ1-3′.

### 2.10. Data Analysis

The significance of differences among various groups was analyzed via Student’s *t*-test in GraphPad Prism software (version 8.4.0), with *p* < 0.05 indicating a statistically significant difference between the two groups (* *p* < 0.05, ** *p* < 0.01, *** *p* < 0.001).

## 3. Results

### 3.1. Schematic of the RAA-CRISPR/Cas12a System for the Detection of PPMV-1

**Figure 1:**
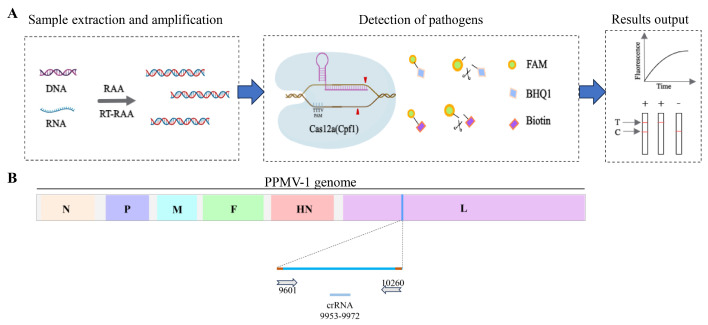
Schematic diagram of RAA-CRISPR/Cas12a-LFD detection and crRNA positioning. (**A**) Schematic diagram of the detection workflow of the RAA-CRISPR/Cas12a-LFD system. (**B**) Schematic diagram of the position of crRNA on the PPMV-1 genome. The crRNA is designed based on the conserved positions of the L gene in the PPMV-1 genome.

In this study, we established a novel strategy for the detection of PPMV-1. As depicted in Figure 1, the detection method relying on RAA-CRISPR/Cas12a involves the following steps: nucleic acid extraction and amplification, specific gene detection, and the resulting output and visualization. During sample preprocessing and amplification, nucleic acids are swiftly extracted from the samples and utilized as templates for RAA. In the detection phase, specifically transcribed and purified crRNA (Figure 1B) is designed to direct Cas12a to identify the target DNA that is complementary to the crRNA. This initiates the cis-cleavage activity of Cas12a toward the target gene and the trans-cleavage activity toward the reporter gene, facilitating subsequent visual detection and onsite testing.

### 3.2. crRNA-Guided CRISPR/Cas12a Cleavage Activity and System Optimization

**Figure 2:**
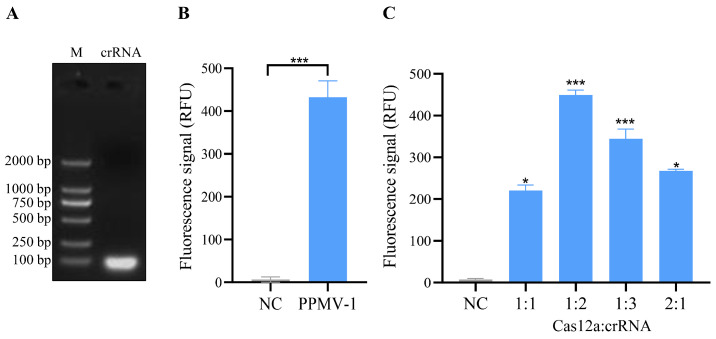
Transcription of crRNA and the detection and optimization of crRNA-mediated CRISPR/Cas12a cleavage activity. (**A**) Electrophoresis results of in vitro-transcribed crRNA. The crRNA template was transcribed in vitro using a T7 transcription kit, followed by purification and electrophoretic analysis. (**B**) Validation of cleavage activity in the CRISPR/Cas12a system. The negative control (NC) utilized nuclease-free water as the template, while the PPMV-1 sample employed PPMV-1 target DNA plasmid as the template. The prepared mixtures were incubated at 37 °C for 10, 20, 30, and 40 min, respectively. A strong fluorescent signal could be detected after 10 min, and the result was presented based on the 10 min reaction. (**C**) Fluorescence intensity of the CRISPR/Cas12a system was measured using various ratios of the Cas12a protein and crRNA. The negative control (NC), in this case, did not include any template. For (**B**,**C**), each sample group contains three replicates. Student’s *t*-test was used for statistical analysis in GraphPad Software Prism 8. *, *p* < 0.5; ***, *p* < 0.001.

On the basis of the PPMV-1 virus sequence alignment results and the design principles of the CRISPR/Cas12a system, a conserved region within the L gene was selected as the target sequence for crRNA design. The strand sequences used for crRNA transcription are shown in Table 1. After the synthesized crRNA strand sequences were annealed, transcription was performed via an in vitro transcription kit. The transcription products were then purified and verified via agarose gel electrophoresis, and the results revealed distinct bright bands (Figure 2A, Figure S1). This finding indicates that the crRNA was successfully transcribed in vitro.

Further analysis was conducted to assess whether the crRNA could guide Cas12a to cleave the PPMV-1 target gene. In this CRISPR/Cas12a system, the crRNA effectively guides the Cas12a protein to perform specific recognition and activate both cis- and trans-cleavage activities. By using PPMV-1 target DNA as a template, specific fluorescence signals can be detected, and the fluorescence signal exhibited a significant difference when compared to the negative control group (Figure 2B). Cas12a and crRNA are key components of this detection system, and their concentration ratio has a significant impact on the detection results. To optimize the CRISPR/Cas12a reaction system, five different concentrations of Cas12a/crRNA were tested. The optimal reaction conditions were achieved at a concentration ratio of the Cas12a protein to a crRNA of 1:2, with concentrations of 50 nM for Cas12a and 100 nM for crRNA (Figure 2C). Under these conditions, the Cas12a/crRNA reactions were analyzed using a real-time fluorescence instrument, resulting in the observation of the strongest fluorescence signal.

### 3.3. Specificity and Sensitivity of the RAA-CRISPR/Cas12a System 

**Figure 3:**
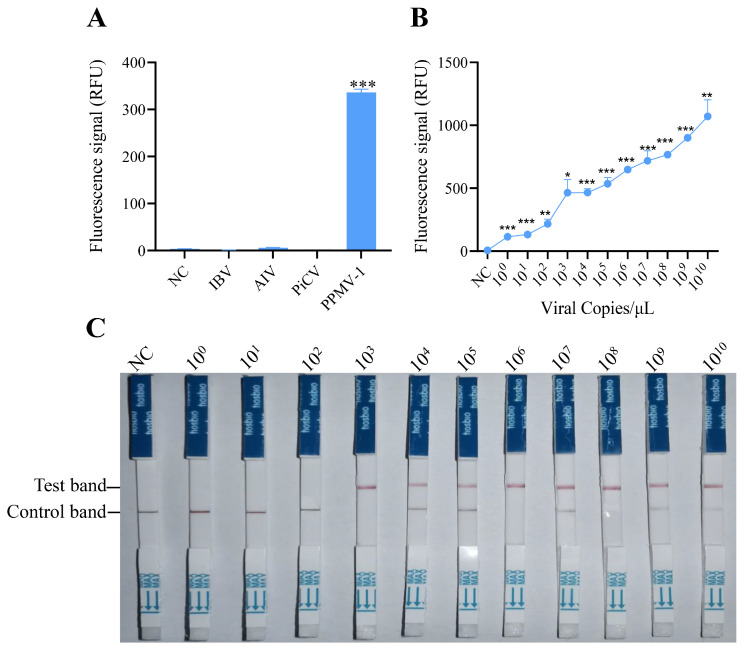
Specificity and sensitivity analysis of the RAA-CRISPR/Cas12a system for PPMV-1 detection. (**A**) Specificity analysis of the RAA-CRISPR/Cas12a system. Fluorescence intensity responding to IBV, AIV, PiCV, and PPMV-1 in the RAA-CRISPR/Cas12a system; NC, negative control (no template). (**B**) Sensitivity analysis of the RAA-CRISPR/Cas12a system. Products of RAA-amplified, serially diluted target DNA were used as templates for the RAA-CRISPR/Cas12a sensitivity analysis. (**C**) Sensitivity analysis of RAA-CRISPR/Cas12a combined with LFD. Data are representative of three independent experiments. Student’s *t*-test was used for statistical analysis in GraphPad Software Prism 8. *, *p* < 0.5; **, *p* < 0.01; ***, *p* < 0.001.

To evaluate the specificity of the RAA-CRISPR/Cas12 system developed in this study, the optimized reaction system was used to detect common pathogens in poultry, including AIV (avian influenza virus), IBV (infectious bronchitis virus), PiCV (pigeon circovirus), and PPMV-1. The fluorescence detection results indicated that specific fluorescence signals were detected only when PPMV-1 was used as the template, and there were no reactions with the genomes of the other pathogens (Figure 3A). These findings demonstrate that the detection method established for PPMV-1 in this study has good specificity.

To assess the sensitivity of detection via the RAA-CRISPR/Cas12a system, serial dilutions of target DNA (ranging from 10^0^ to 10^10^ copies) were tested. These templates were first amplified via RAA and then analyzed for sensitivity via the CRISPR/Cas12a detection system. The fluorescence signals were collected every 2 min, with three replicates per reaction. Fluorescence intensity analysis revealed that target DNA could be effectively detected at a concentration of 10^0^ copies/μL (Figure 3B), which suggests that the RAA-CRISPR/Cas12 system has excellent sensitivity for detecting PPMV-1.

To determine the sensitivity of the established RAA-CRISPR/Cas12a system combined with a lateral flow dipstick for detecting PPMV-1, diluted target DNA (with concentrations ranging from 10^0^ to 10^10^ copies/µL) was used as the template. Following RAA and the CRISPR/Cas12a reaction, the CRISPR Cas12/13 HybriDetect test strip was immediately inserted. The results after 2 min indicated that a clear T line could be observed when the target DNA concentration was 10^3^ copies/μL (Figure 3C). These findings demonstrate that the RAA-CRISPR/Cas12a-LFD system can be effectively used for the detection of PPMV-1.

### 3.4. Onsite Detection Capability of RAA-CRISPR/Cas12a-LFD 

**Figure 4:**
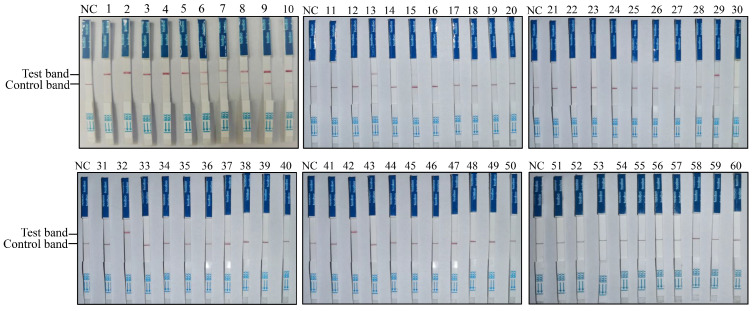
Detection of clinical pigeon throat and cloacal swab samples using the RAA-CRISPR/Cas12a-LFD technique. RNA extracted from 60 throat and cloacal swab samples of pigeons was used as the template for RAA-CRISPR/Cas12a-LFD detection.

To establish the RAA-CRISPR/Cas12a-LFD-based PPMV-1 detection method, this approach was applied to clinical sample detection. Sixty pigeon throat and cloacal swabs from Shanxi Province (China) were collected and tested for PPMV-1 infection. The results obtained with RAA-CRISPR/Cas12a-LFD for the 60 clinical swab samples are shown in Figure 4; 14 out of the 60 samples were positive for PPMV-1 (Figure 4, Table 2).

**Figure 5:**
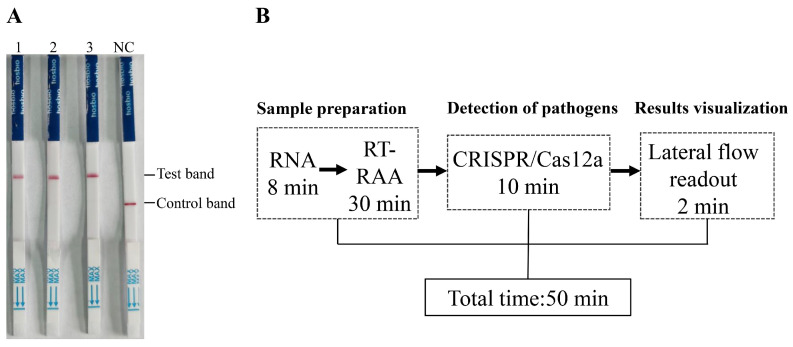
Detection of tissue samples in diseased pigeons and the timeline for the RAA-CRISPR/Cas12a-LFD assay. (**A**) The detection results of lung tissue homogenates from three diseased pigeons. (**B**) Schematic diagram of the total time and duration of each step for detection using RAA-CRISPR/Cas12a-LFD.

To assess the effectiveness and reliability of the RAA-CRISPR/Cas12a-LFD method in clinical applications, the results were compared with those obtained via traditional PCR and real-time quantitative PCR (qPCR) methods using the same clinical swab samples. A total of 14 samples were found to be positive by both the RAA-CRISPR/Cas12a-LFD method and qPCR, and there was a total of 8 positive samples that were also detected with traditional PCR (Table 2, Table S1). The detailed detection results of the 60 clinical samples revealed that the concordance rate of RAA-CRISPR/Cas12a-LFD with conventional PCR results was (8 + 46)/60 = 90%, while the concordance rate between RAA-CRISPR/Cas12a-LFD and qRT-PCR results was (14 + 46)/60 = 100% (Table S1). These results indicate that, compared with conventional PCR, the RAA-CRISPR/Cas12a-LFD method has superior detection performance in complex clinical swabs. The positive detection rate of the RAA-CRISPR/Cas12a-LFD method was consistent with that of qPCR. The RAA-CRISPR/Cas12a-LFD method was also applied to tissue samples positive for PPMV-1. All three PPMV-1-positive tissue samples tested yielded positive results via the RAA-CRISPR/Cas12a-LFD method (Figure 5A). Additionally, we utilized both a handheld electric tissue grinder and liquid nitrogen to homogenize four different tissue samples (trachea, lungs, brain, and liver) before conducting subsequent RAA-CRISPR/Cas12a-LFD detection. We found that both grinding methods did not affect the specificity and sensitivity of the detection results, and the handheld grinder proved to be more convenient for onsite testing (Figure S2). This finding fully demonstrates the accuracy and reliability of the RAA-CRISPR/Cas12a-LFD method in detecting PPMV-1 in tissue samples.

In terms of detection time, the established RAA-CRISPR/Cas12a-LFD method requires only 50 min to complete the entire detection process (Figure 5B). This rapid turnaround time highlights the method’s potential utility for timely and efficient PPMV-1 detection in clinical settings, offering a reliable and efficient alternative to traditional PCR and qPCR methods.

## 4. Discussion

PPMV-1, an antigenic variant of NDV, has a clear host preference for pigeons and has caused substantial economic losses in the poultry industry worldwide [6,20]. In China, PPMV-1 was first identified in 1985 and has since spread nationally, posing significant threats to the health of pigeon populations [21]. Among the various sub-genotypes, Sub-genotype VI has emerged as the predominant strain in recent years, particularly within Chinese pigeon flocks [22]. Our study identified all positive samples from clinical testing as belonging to the VI.2.1.1.2.2 sub-genotype, which is consistent with recent research findings [22,23]. While most PPMV-1 isolates do not cause significant clinical manifestations in chickens, they demonstrate high pathogenicity in pigeons [24,25,26]. Some studies have reported that NDV strains originating from pigeons can also exhibit high pathogenicity in chickens, highlighting the necessity for prompt monitoring and the pathogenicity assessment of pigeon-derived NDV strains [27,28]. Although PPMV-1 infection is endemically prevalent in domestic pigeons, it can also occur in wild doves, other bird species, and even humans, leading to severe clinical symptoms and potentially fatal outcomes [29,30]. Therefore, the rapid diagnosis and control of PPMV-1 are crucial for the pigeon industry and public health safety.

Currently, the commonly used detection methods for PPMV-1 include virus isolation and identification, HA-HI testing, ELISA testing, RT–PCR, and real-time PCR [7,8,21]. However, these methods have limitations, such as long detection times, the requirement for expensive equipment, complex operation procedures, and the need for specialized technical personnel. Loop-mediated isothermal amplification (LAMP) has been developed for the rapid detection of NDV and offers significant advantages in terms of convenience, speed, and sensitivity [31,32]. However, LAMP has several drawbacks, including the complexity of primer design, the risk of contamination, and the possibility of nonspecific amplification. Owing to the small-scale nature of most pigeon or racing pigeon breeding farms with limited detection conditions, there is an urgent need for the rapid, easy-to-use, highly specific, and sensitive detection method for PPMV-1.

Recently, a blocking lateral flow assay (bLFA) strip was developed for the rapid clinical detection of NDV antibodies, and this method allows for the detection of Newcastle disease-specific antibodies at room temperature in 10 min, and it demonstrates high specificity and sensitivity [33]. However, the rapid and precise detection of pathogens is also of significant importance for the prevention and control of Newcastle disease in pigeons. In the present study, we developed an RAA-CRISPR/Cas12a-LFD assay, which is simple, rapid, and accurate for the detection of PPMV-1. Unlike LAMP and qRT–PCR, our method does not require high temperatures or expensive equipment, and the reaction can be completed in just 50 min. Furthermore, the integration of CRISPR technology enhances the specificity of our method because of its precise gene-editing capabilities. Moreover, the combined utilization of LFD transforms the detection method into a simple and visual assay, making it ideal for convenient onsite clinical testing. The RAA-CRISPR/Cas12a-LFD detection method we developed has demonstrated promising results for identifying the sub-genotype VI.2.1.1.2.2 of PPMV-1. However, it is crucial to investigate whether this detection method can be effectively applied to other genotypes of PPMV-1 in the future. In addition, there is a need to further optimize sample processing and rapid nucleic acid extraction methods. Streamlining these procedures will facilitate the rapid application of our detection method in field settings, making it more accessible for onsite testing. The ability to quickly and accurately detect PPMV-1 is vital for epidemiological investigations, monitoring outbreaks, and implementing timely interventions.

## 5. Conclusions

We developed a novel method that combines RAA-CRISPR/Cas12a with LFD for the detection of PPMV-1 in pigeons. This method offers several advantages, including ease of operation, cost-effective equipment, a short experimental time, and high specificity and sensitivity. This method shows significant potential for clinical applications and can be used for the onsite testing and routine monitoring of PPMV-1 in pigeon flocks.

## Figures and Tables

**Table 1:** The crRNA, primers, and probes used in this study.

Primer Name	Sequence (5′→3′)
PPMV-1-crRNA-F	GAAATTAATACGACTCACTATAGGGTAATTTCTACTAAGTGTAGATTAGAATCAAATGATTTTGAT
PPMV-1-crRNA-R	ATCAAAATCATTTGATTCTAATCTACACTTAGTAGAAATTACCCTATAGTGAGTCGTATTAATTTC
PPMV-1-Target DNA-F	CCATGGGAAGAAGAATTCAGGT
PPMV-1-Target DNA-R	CTCGAGTCATGATTGCGGTTT
PPMV-1-RAA-F	AACCTCAACTAACCGCCTCTTGATAGAGTTT
PPMV-1-RAA-R Reporter I Reporter II	CTGCCATTACCTGGCAGTTTCTTAATCT FAM-TTATTATT-BHQ1 FAM-TTATTATT-Biotin

**Table 2:** Comparison of clinical sample detection results between RAA-CRISPR/Cas12-LFD and conventional PCR and qPCR.

Detection Results	PCR (Positive/Total)	qPCR (Positive/Total)	RAA-CRISPR/Cas12a-LFD (Positive/Total)
Number of positive samples	8/60	14/60	14/60
Positive rate	13%	23%	23%

## Data Availability

Data are contained within this article and Supplementary Materials.

## Supplementary Materials

The following supporting information can be downloaded at https://www.mdpi.com/article/10.3390/vetsci11100473/s1. Figure S1: Original gel image of Figure 2A. Figure S2: RAA-CRISPR/Cas12a-LFD detection of the same tissue samples processed using either a handheld electric tissue grinder or liquid nitrogen. Table S1: Detailed information of 60 clinical samples and the corresponding detection results for the three methods.
