# Supplementary material for: RAA-CRISPR/Cas12a-Mediated Rapid, Sensitive, and Onsite Detection of Newcastle Disease in Pigeons
Source: Vet Sci. 2024 Oct 4;11(10):473. doi: 10.3390/vetsci11100473 (PMC11512335; doi:10.3390/vetsci11100473)
Supplement: Supplementary file 1 [file vetsci-11-00473-s001.zip › vetsci-3201799-supplementary.pdf]

Supplementary Table S1. Detailed information of 60 clinical samples and the corresponding detection results for the three methods.

Sample number	Sample type (throat swab/ cloacal swab	PCR (+/-)	qPCR (+/-)	RAA CRISPR/Cas12a -LFD (+/-)
1	throat	+	+	+
2	throat	+	+	+
3	cloacal	-	+	+
4	throat	+	+	+
5	throat	+	+	+
6	cloacal	-	+	+
7	throat	-	+	+
8	throat	-	+	+
9	throat	+	+	+
10	cloacal	+	+	+
11	throat	-	-	-
12	cloacal	-	-	-
13	throat	-	+	+
14	throat	-	-	-
15	cloacal	-	-	-
16	throat	-	-	-
17	throat	-	-	-
18	cloacal	-	-	-
19	cloacal	-	-	-
20	cloacal	-	-	-
21	cloacal	-	-	-
22	throat	-	-	-
23	cloacal	-	-	-
24	throat	-	-	-
25	throat	-	-	-
26	cloacal	-	-	-
27	throat	-	-	-
28	cloacal	-	-	-
29	cloacal	+	+	+
30	throat	-	-	-
31	cloacal	-	-	-
32	cloacal	-	+	+
33	throat	-	-	-
34	cloacal	-	-	-
35	throat	-	-	-
36	cloacal	-	-	-
37	cloacal	-	-	-
38	cloacal	-	-	-

39	throat	-	-	-
40	cloacal	-	-	-
41	Throat	-	-	-
42	Throat	+	+	+
43	cloacal	-	-	-
44	throat	-	-	-
45	cloacal	-	-	-
46	throat	-	-	-
47	throat	-	-	-
48	throat	-	-	-
49	cloacal	-	-	-
50	cloacal	-	-	-
51	cloacal	-	-	-
52	throat	-	-	-
53	cloacal	-	-	-
54	cloacal	-	-	-
55	throat	-	-	-
56	cloacal	-	-	-
57	throat	-	-	-
58	throat	-	-	-
59	cloacal	-	-	-
60	throat	-	-	-

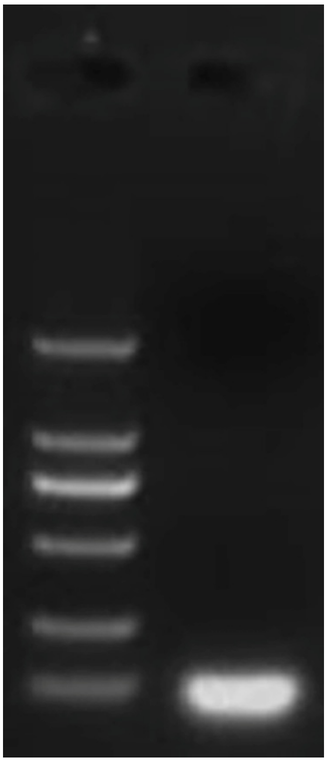

Supplementary Figure S1. Original gel image of Figure 2A. Electrophoresis results of in vitro-transcribed crRNA. The crRNA template was transcribed in vitro using a T7 transcription kit, followed by purification and electrophoretic analysis.

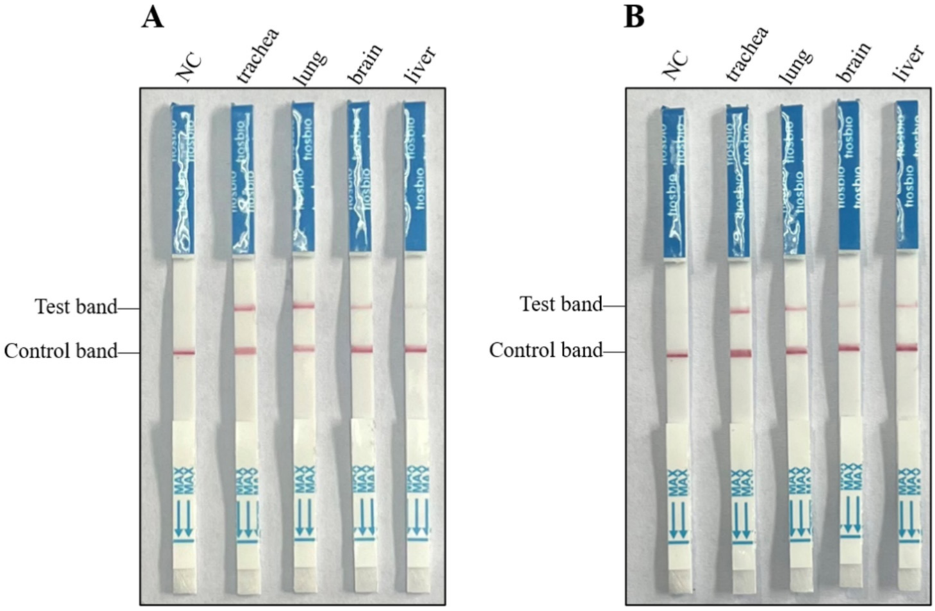

Supplementary Figure S2. RAA-CRISPR/Cas12a-LFD detection of the same tissue samples processed using either a handheld electric tissue grinder or liquid nitrogen. (A) RAA-CRISPR/Cas12a-LFD detection results for tissue samples obtained from the trachea, lung, brain, and liver of diseased pigeons following digestion with a handheld grinder. (B) RAA-CRISPR/Cas12a-LFD detection results for tissue samples from the trachea, lung, brain, and liver of diseased pigeons after digestion using liquid nitrogen grinding.
